# Supplementary material for: From sequence to enzyme mechanism using multi-label machine learning
Source: BMC Bioinformatics. 2014 May 19;15:150. doi: 10.1186/1471-2105-15-150 (PMC4229970; doi:10.1186/1471-2105-15-150)
Supplement: Additional file 2 — Java code of ml2db. Additional file ml2db_code.tar.gz contains the Java source code to run the multi-label machine learning experiments and save the results to database. The code’s Javadoc is included. [file 1471-2105-15-150-S2.zip › additional file 2/ml2db/READ_ME.pdf]

ml2db manual

Luna De Ferrari ldf@st-andrews.ac.uk

April 30, 2014

The ml2db project code (also called EnzML in some of our publications) supports the generation of Mulan ARFF (attribute relation file format) files from database data. It also facilitates machine learning experiments (cross-evaluation, training-testing evaluation and class predictions), saving all evaluation and prediction results to database.

The suggested code development and running environment is Eclipse https://www.eclipse.org/, but examples for command line running are provided.

Data between <> brackets should be customised according to your file names, file paths, user name etc.

The set up of a machine learning experiment is summarised below.

Generate or choose a data file

- If the ARFF/Mulan data file with your data of interest has already been generated, just input its path or reuse its database identifier (taken from the *arff* database table)
- If the data file doesn't exist yet:
  1. *First step (one off): set the database connection to the database where the data is stored*
  2. Write in a .props file the SQL queries to select the data of interest (a list of instance+attribute and another list of instance+class label)
  3. Generate the data file with the MulanArff class

Cross-validation

1. *First step (one off): set the database connection to the database where the results should be saved*
2. Generate or choose a data file
3. Choose
   - an algorithm,
   - the number of folds
   - whether to save predictions to database or not
4. run a cross evaluation over that data file

Training-testing evaluation

1. *First step (one off): set the database connection to the database where the results should be saved*
2. Generate of choose one data file for training and one for testing
3. Choose
   - an algorithm
   - whether the training and testing files need filtering to become compatible (not necessary if they have the same attributes and classes)
   - whether to save predictions to database or not
4. run a training-testing evaluation over the training and test data files

1 To create a data file

1.1 Set the database connection

To create a data file, you must first set the connection to the input (data) and output (evaluation and predictions) databases (***only for the first use).***

The databases must already exist and the user specified must have read and write permissions. Make sure the jdbc database driver is in the Eclipse project Java path. You can then customise the database connection details under *ml2db/data/main/properties_files/machine_learning_properties/*, where two files must be customised:

1. the text file *mulan_data_local_server.props* with the connection details of the input database (where the data will be found)
2. the text file *mulan_results_local_server.props* with the connection details of the outptu database (where the ARFF file records, evaluation and predictions should be saved).

An example content for a MySQL database would be:

host=localhost
port=3306
database_name=<mydatabase>
user=<myuser>
pswd=<mypassword>
database_type=mysql
jdbc_driver=org.gjt.mm.mysql.Driver

1.2 Create a data .props file

To collect the data you want in the data file, write some SQL queries and the dataset name of your choice, in a .props text file under:

- ml2db/data/main/properties_files/arff_props/<my_file>.props

Example content:

arff_file_path=data/test/arff/
dataset=<instances_1_2>
description=<Two instances for test purposes.>
attributesQuery=<SELECT DISTINCT instance, attributeValue FROM instanceAttributeTable>
instance_class_query=<SELECT DISTINCT instance, class FROM instanceClass>

xml_query=<SELECT DISTINCT class FROM instanceClass>

The *xml_query* is an essential, separate query to get exclusively the class labels. It is generally very similar to the *instance_class_query* but with only the class column selected. It can be used under special circumstances to inject additional classes or for testing purposes and should not be left empty.

1.3 Create the ARFF and XML files

Finally, you can create your data files. Mulan stores the data in a Weka style ARFF file, where both the class labels and the feature attributes are marked as *@Attribute*. To distinguish which attributes are class attributes (labels), Mulan also needs an XML file with the class labels are stored either in a flat or hierarchical style. (For improving human reading, in our format the name of feature attributes starts with 'FA_' and the name of class attributes starts with 'CA_' but this information is not recognised by Mulan).

To generate the Mulan ARFF and XML files, launch the MulanArff Java class main.

Input If the classpath is correctly set, the following command should generate your files, when launched *from the folder 'ml2db'* (the one containing the folders 'src', 'doc', 'data' , 'bin' etc.):

- *java main.ml2db.mulan.mulanarff.MulanArff -arffqueries data/main/properties_files/arff_props/<my_data_querie*

Or you can include the path to reach all needed jars:

- */usr/lib/jvm/jdk1.7.0/bin/java -Xms2500M -Xmx2500M -Dfile.encoding=UTF-8 -classpath /<path_to_eclipse_hon 0900/junit.jar:/usr/local/share/eclipse/plugins/org.hamcrest.core_1.1.0.v20090501071000.jar:/<path_to_eclipse_ 4.8.2.jar:/<path_to_eclipse_home>/jars/Tidy.jar:/<path_to_eclipse_home>/jars/mysql-connector-java-5.1.12-bin.jar:/<path_to_eclipse_home>/jars/jdom.jar:/<path_to_eclipse_home>/jars/apache-log4j-1.2.16/log4j-1.2.16.jar:/<path_to_eclipse_home>/ecmulan/bin:/<path_to_eclipse_home>/jars/weka-3.7.6.jar:/<path_to_eclipse_home>/mulan_14/bin main.ml2db.mulan.mulanarff.MulanArff -arffqueries /data/main/properties_files/arff_props/file_with_my_data_queries.props*

Or you can launch MulanArff.java with an IDE such as Eclipse, adding the option *-arffqueries data/main/properties_files/a* to the run configuration.

Output One ARFF file (for the data) and one XML file (for the class labels) will be created under:

- ml2db/data/main/arff/<my_dataset_name>_<timestamp>.arff and <my_dataset_name>_<timestamp>.xml

A record will also be written into the table <my_results_database>.arff. If the arff table does not exist, it will be created. The database record contains a unique identifier for the ARFF dataset, the dataset name, the queries, a timestamp, some data statistics (number of instances, class labels and attributes) and the full ARFF data (for files smaller than 1GB).

1.4 Get hold of the ARFF identifier for future use

The ARFF+XML datafile needs to be created only once. To use the data file for further analysis in ml2db, get hold of its identifier (an integer number) either from the screen log when creating the file or from the arff database table.

An example SQL query to get the last data file created would be: *select max(id) from <my_results_db>.arff;*

1.5 Alternative settings

The standard location of the ARFF and XML files, data .props files and database .props file can be changed in the Java class main.ml2db.ProjectParameters. Leave the ARFF and XML files in that location if you want to be able to run experiments calling those files by their identifier.

2 Cross-evaluation and prediction

One ARFF+XML data file can be used for cross-evaluation, where the data is partitioned into folds, which are then used as training or testing data in a collection of independent experiments.

This is an example of command to launch 20 repetitions of a 10-fold cross-evaluation on data file 411, saving the predictions to database (not saving the True Negatives nor stratifying the data):

- /usr/lib/jvm/jdk1.7.0/bin/java main.ml2db.mulan.learn.crosseval.CrossExperimenter -arffid 411 -savepredictions -folds 10 -evaluations 20

Or, with full classpath:

- /usr/lib/jvm/jdk1.7.0/bin/java -Xms2600M -Xmx2600M -Dfile.encoding=UTF-8 -classpath /<path_to_code>/ml2
0900/junit.jar:/usr/local/share/eclipse/plugins/org.hamcrest.core_1.1.0.v20090501071000.jar:/<path_to_code>/u
4.8.2.jar:/<path_to_code>/jars/Tidy.jar:/<path_to_code>/jars/mysql-connector-java-5.1.12-
bin.jar:/<path_to_code>/jars/jdom.jar:/<path_to_code>/jars/apache-log4j-1.2.16/log4j-1.2.16.jar:/<path_to_
3.7.6.jar:/<path_to_code>/mulan_14/bin main.ml2db.mulan.learn.crosseval.CrossExperimenter
-arffid 411 -savepredictions -folds 10 -evaluations 20

The options in more detail:

arfffileid an ARFF file identifier in the arff database table

learners (in the main method) a vector of algorithms. See the MulanLearners class for more examples

savepredictions whether predictions should be saved to database. If so, a master record will be created in the <my_results>.prediction_experiments table and each instance-class prediction will be stored in the <my_results>.prediction_records table in the results/output database of you chose (see *Set the database connection* section above)

savetruenegatives whether True Negative prediction should be saved to database. It should be set to false if the classes are many and sparse, because it can be computationally intensive and not particularly informative

stratify whether the cross-evaluation should stratify the data first, that is, instead to separating the data randomly into folds, to equally distribute the data into folds according to their class labels

folds the number of folds. Typical values are 10 (for 10-fold cross-validation) or -1 to have as many folds as instances (leave-one-out validation).

evaluations for non-leave-one-out experiments the folds are randomly picked, which could cause inbalance in the data. Setting the numberOfCrossEval > 1 runs the entire cross-validation experiment (including the fold randomisation) a number of times (for example 10 or 20). The resulting evaluation results can then be averaged, the standard deviation calculated etc.

Several files and algorithms can be run at once by editing the code. If the cross-evaluation must be repeated with **multiple algorithms**, they can be added as lines such as: *learners.add(MulanLearners.getBRk1NN());* in the main method of the class main.ml2db.mulan.learn.crosseval.CrossExperimenter.

3 Training-testing evaluation and prediction

A training-testing evaluation (training on one dataset and testing on a separate dataset) can be launched from the Java class *main.ml2db.mulan.learn.traintest.TrainTestFullRun*.

The data files for training and test must be compatible, that is, must have the same attributes and classes. The files can be made compatible beforehand by running the method *AttFilter.oneFilterRun()*

or directly during the training+testing experiment. When filtering, one file is considered the "master" training set, and the other the testing set. If you want to change the role of a file (for example to use it as a test set instead of training set) the files must be filtered anew.

After filtering, a new arff record (and ARFF and XML file) will be generated for the new, compatible training data and another for the new, compatible test set. The naming of the new files will include the arff identifiers of the original training and testing data files, for example: *ml2db/data/main/arff/filt_7_with_attr_of_5_a* (what a mouthful, I know...) indicates a file containing the data of the arff with id 7, which has been made compatible with the arff file with id 5. The file was generated on the 3rd of Feb 2014 at time 19:22:46 and 494 microseconds.

An example of command training on data file 21, testing on data file 22 and saving predictions (including the true negative predictions) to database (the data files are considered not compatible and hence they will be filtered):

- java main.ml2db.mulan.learn.traintest.TrainTestFullRun -trainingarffid 21 -testarffid 22 -predict -savetruenegatives

With full classpath:

- /usr/lib/jvm/jdk1.7.0/bin/java -Xms600M -Xmx2600M -Dfile.encoding=UTF-8 -classpath /<your_path_to_code> 0900/junit.jar:/usr/local/share/eclipse/plugins/org.hamcrest.core_1.1.0.v20090501071000.jar:/<your_path_to_co 4.8.2.jar:/<your_path_to_code>/jars/Tidy.jar:/<your_path_to_code>/jars/mysql-connector-java-5.1.12-bin.jar:/<your_path_to_code>/jars/jdom.jar:/<your_path_to_code>/jars/apache-log4j-1.2.16/log4j-1.2.16.jar:/<your_path_to_code>/ecmulan/bin:/<your_path_to_code>/jars/weka-3.7.6.jar:/<your_path_to_code>/mulan_14/bin main.ml2db.mulan.learn.traintest.TrainTestFullRun -trainingarffid 21 -testarffid 22 -predict -savetruenegatives

The command options in more detail:

alreadyfiltered to be set if the files are already compatible; if excluded, the files will be filtered. To avoid filtering multiple times, run the first experiment without the '-alreadyfiltered' option and then get from the database *arff* table the identifiers of the filtered arffs to use for subsequent experiments (which can be run without the option '-alreadyfiltered').

predict whether predictions should be saved to database. If so, a master record will be created in the <my_results>.prediction_experiments table and each instance-class prediction will be stored in the <my_results>.prediction_records table in the results/output database of you chose (see *Set the database connection* section above) .

savetruenegatives whether True Negative prediction should be saved to database. It should be avoided if the classes are many and sparse, because it can be computationally intensive and not particularly informative.

trainingarffid the arff table record identifier for the training set

testarffid the arff table record identifier for the test set

learner (in the main method code) the chosen algorithm. It can be customised in the MulanLearners class, which contains several examples of Mulan multi-label algorithms and Weka multi-class algorithms wrapped to make them multi-label.

4 Results

ARFF data file records are saved in the table <my_output_database>.arff

Evaluation results (accuracy, precision, recall, number of false positives etc.) are saved to the table <my_output_database>.mulan_results

Predictions are saved in tables: prediction_experiments (one master record per prediction experiment, specifying the start time, training arff id, testing arff id etc.) and prediction_records (one row per instance-class prediction). If the test data file includes the true class labels, the prediction row will also include the prediction error status (false negative, false positive, true positive, or true negative, if true negatives have set to "save").
